# Marine Seaweed Polysaccharides-Based Engineered Cues for the Modern Biomedical Sector

**DOI:** 10.3390/md18010007

**Published:** 2019-12-19

**Authors:** Muhammad Bilal, Hafiz M. N. Iqbal

**Affiliations:** 1 School of Life Science and Food Engineering, Huaiyin Institute of Technology, Huaian 223003, China; 2 Tecnologico de Monterrey, School of Engineering and Sciences, Campus Monterrey, Ave. Eugenio Garza Sada 2501, Monterrey 64849, Mexico

**Keywords:** marine seaweed, polysaccharides, engineered cues, nanocarriers, drug delivery, wound healing, biomedical applications

## Abstract

Seaweed-derived polysaccharides with unique structural and functional entities have gained special research attention in the current medical sector. Seaweed polysaccharides have been or being used to engineer novel cues with biomedical values to tackle in practice the limitations of counterparts which have become ineffective for 21st-century settings. The inherited features of seaweed polysaccharides, such as those of a biologically tunable, biocompatible, biodegradable, renewable, and non-toxic nature, urge researchers to use them to design therapeutically effective, efficient, controlled delivery, patient-compliant, and age-compliant drug delivery platforms. Based on their significant retention capabilities, tunable active units, swelling, and colloidal features, seaweed polysaccharides have appeared as highly useful materials for modulating drug-delivery and tissue-engineering systems. This paper presents a standard methodological approach to review the literature using inclusion-exclusion criteria, which is mostly ignored in the reported literature. Following that, numerous marine-based seaweed polysaccharides are discussed with suitable examples. For the applied perspectives, part of the review is focused on the biomedical values, i.e., targeted drug delivery, wound-curative potential, anticancer potentialities, tissue-engineering aspects, and ultraviolet (UV) protectant potential of seaweed polysaccharides based engineered cues. Finally, current challenges, gaps, and future perspectives have been included in this review.

## 1. Introduction

Over the past four decades, the biomedical sector at large and drug-delivery platforms in particular, have witnessed significant advancements in this domain of the 21st-century world. Throughout this venture, research scientists, both from the academia and industrial sector, have revisited and adapted their efforts and focus on (re)searching and engineering materials and cues/carriers with potentialities to meet the rising demands of medical and health care settings. The drug-delivery perception necessitates the precise transfer of a specific dose of numerous therapeutic cues and biologically active agents to the desired site within a programmed/coded period [1,2,3,4]. The short life span of drugs in a natural body environment, inefficient delivery, burst release, safer way out, and complications of treatment sites, such as tumor sites, are major challenges faced by several drug delivery systems in practice [5,6]. Additionally, futher complications arise when the drug is overused or misused which leads to the diseases recurrence and ultimately acquire resistance. Thus, aiming to ensure the safety and overall effectiveness, new engineered cues using naturally-occurring seaweed polysaccharides with inherited biomedical values are being developed and exploited as a combinational approach. This is because the seaweed polysaccharides based engineered carriers can overcome the aforementioned challenges as well as improve the stability and solubility of encapsulated drugs. Considering these points, seaweed polysaccharides-based engineered carriers provide an opportunity to (re)evaluate the overall treatment efficacy of numerous drug candidates which have been mostly abandoned due to poor pharmacokinetics or a failure to fulfill the therapeutic window [7].

**Figure 1:**
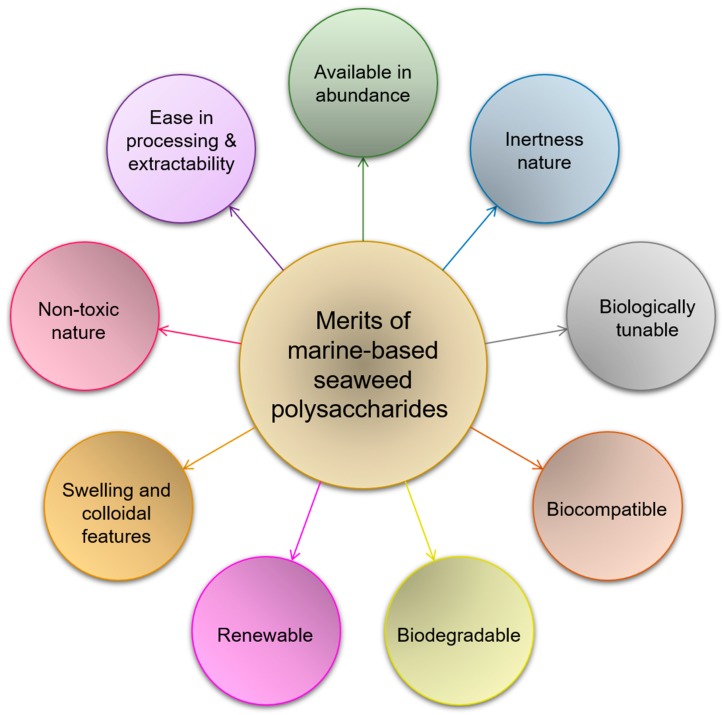
Schematic illustration of notable merits, unique structural and functional entities of marine-based seaweed polysaccharides.

**Figure 2:**
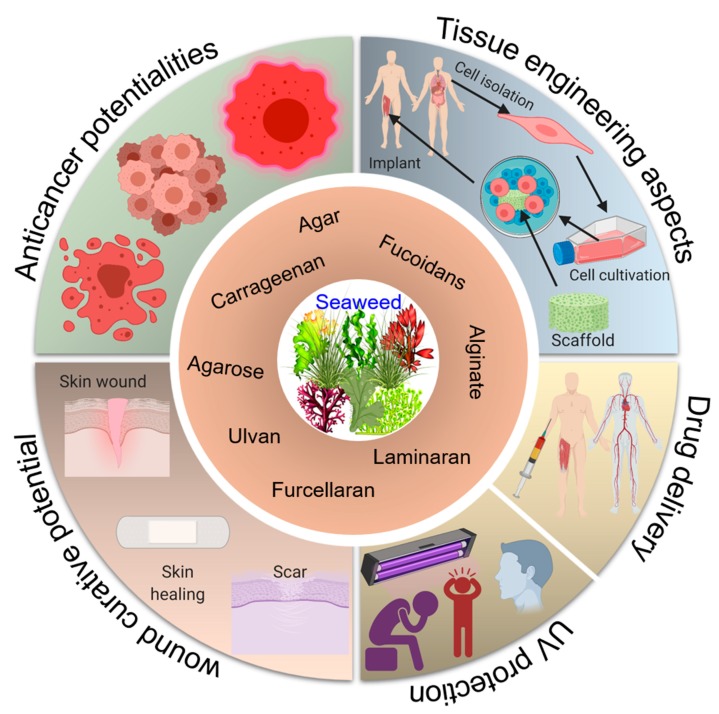
Biomedical potentialities of marine-based seaweed polysaccharides, i.e., carrageenan, ulvan, fucoidans, alginate, agar, agarose, laminaran, and furcellaran.

This paper presents a standard methodological approach to review the literature using inclusion-exclusion criteria, which is mostly ignored in the reported literature belongs to this domain, i.e., seaweed-derived polysaccharides. Herein, we first review several seaweed-derived polysaccharides, such as carrageenan, ulvan, fucoidans, alginate, agar, laminaran, and furcellaran. Their unique structural and functional entities are discussed with suitable examples (Figure 1). The latter half of the review focuses on biomedical perspectives of seaweed-derived polysaccharides. For instance, targeted drug delivery, wound-curative potential, anticancer potentialities, tissue engineering aspects, and ultraviolet (UV) protectant potentialities (Figure 2) are discussed and illustrated with representative action mechanisms. Towards the end, numerous challenges, research gaps, concluding remarks, and future recommendations or outlooks are also given to pave the way for further studies in this important domain.

## 2. Methodological Approach—Inclusion/Exclusion Criteria

**Figure 3:**
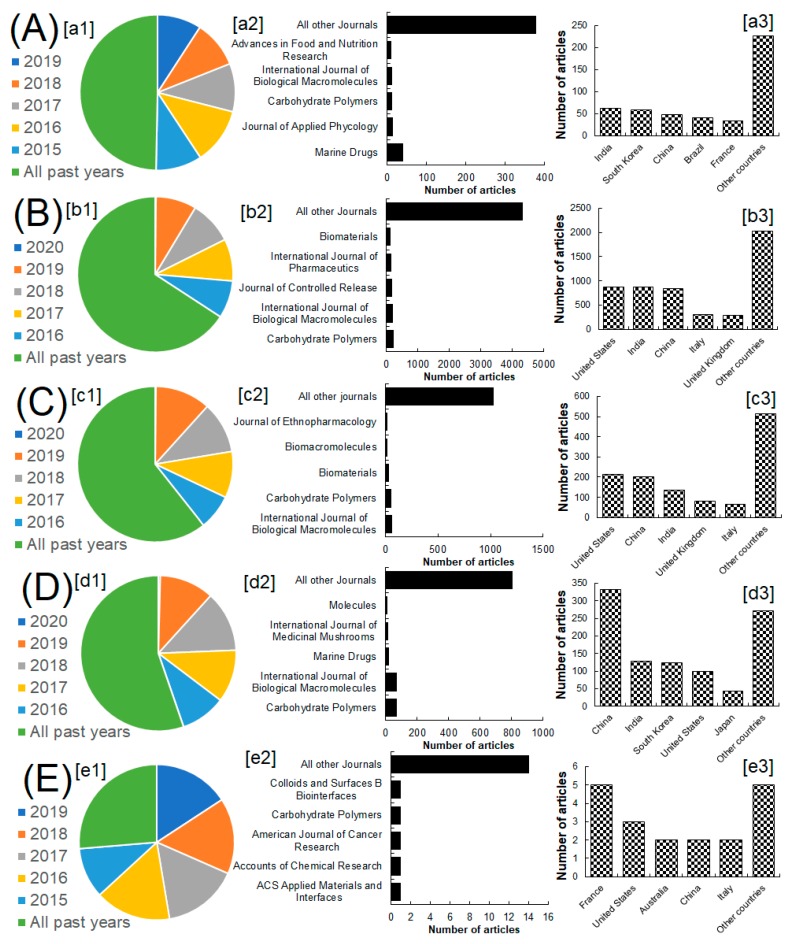
Literature search results obtained from the Scopus database. The capital letters (**A**–**E**) represent the search terms, i.e., (**A**) Marine seaweed polysaccharides, (**B**) Drug delivery using polysaccharides, (**C**) Wound healing using polysaccharides, (**D**) Anticancer activities of polysaccharides, and (**E**) Polysaccharides based engineered carriers. The lower letters [a1–e1] represents the number of articles from all years in that specific category of search term, [b2–e2] represents the number of articles published in different journal, and [a3–e3] represents the number of articles based on territory. For detailed numbers, please see the Supplementary Table (Table S1) at the end.

A standard methodological approach to review the literature using inclusion-exclusion criteria, which is mostly ignored in the reported literature, was adopted. This was also aimed at justifying the following points, i.e., (1) the scientific theme of the work, and (2) to cover the recent and relevant literature content. For this purpose, the authentic scientific literature databases, such as Scopus, and PubMed were used to perform the literature survey. The collected literature data was carefully analyzed ensuing the inclusion-exclusion criteria, the studies closely matched with the title theme were included unless otherwise excluded. For this purpose, an initial literature screening was performed based on the presence of all or any of the following key terms, i.e., marine seaweed polysaccharides; drug delivery using polysaccharides, wound healing using polysaccharides, anticancer activities of polysaccharides, and polysaccharide-based engineered carriers, in the article title, abstract and keywords. Figure 3 summarizes the search results obtained from the Scopus database. The literature search queries were performed on 21 November 2019, at https://www.scopus.com. In PubMed, the literature was searched for all published years with the best match term on. The search results obtained from the PubMed database are summarized in Table 1. Considering the best match terms, i.e., marine seaweed polysaccharides, drug delivery, wound healing, and anticancer activities of polysaccharides and polysaccharide-based engineered carriers, the inclusion-exclusion criteria were applied for initial screening. During the initial screening, any type of article that discussed the above-selected search terms were only considered relevant to the scope of this review and thus included accordingly. Further exclusion was considered if none of the searched term was dealt with and discussed in the results section for articles. Based on the literature data obtained, the following sections and subsections were conceptualized and discussed with suitable examples as a core of this review.

## 3. Marine Seaweed Polysaccharides

Naturally occurring bioactive compounds and metabolites are regarded as safe for human health and thus have been widely utilized in folk medicines to cure many illnesses. The proper utilization of such compounds has gained special interest to ensure maximum health-related benefits. Amongst the various useful bio-constituents, polysaccharides obtained from seaweed have shown numerous beneficial biological functions such as antioxidant, anticarcinogenic, antiviral, targeted drug-delivery, wound-curative and anti-inflammatory activities. A vast number of recent reports have revealed the potential biomedical and pharmaceutical potentialities of polysaccharides extracted and purified from various seaweeds. In this section, different seaweed-derived polysaccharides, and their structure and biological activities are discussed.

### 3.1. Carrageenan—Structural and Functional Entities

Carrageenan is a marine-derived anionic sulfated polysaccharide comprising alternating *β*-d-galactose and *α*-d-galactose or 3,6-anhydro-*α*-d-galactose residues, which are connected with each other through *α*-1,3 and *β*-1,4-glycosidic linkages. It is the major constituent of the cell wall of several red seaweeds families, such as *Eucheuma denticulatum*, *Kappaphycus alvarezii*, *C. crispus*, and *Gigartina* species [8,9]. Based on sulfate content and position, carrageenans are classified into six different types including Lambda-CG (*λ*), Theta-CG (*θ*), Iota-CG (*ι*), Kappa-CG (*κ*), Mu-CG (*μ*), and Nu-CG (*ν*). Lambda, Kappa, and Iota are the most significant and commercialized forms among the six types of CG and are different from each other based on the number of sulfate groups [10]. Kappa-CG contains one and two ester sulfate groups per repeating disaccharide unit, where *λ*-CG has three sulfate groups on every disaccharide unit that constitute up to 40% (*w/w*) of sulfate content [11]. Differences in sulfate contents are imperative for the interactive and functional properties of various carrageenans [12]. Biologically, Nu-CG and Mu-CG are formed from Iota- and Kappa CG, respectively, whereas Lambda-CG gives rise to theta-CG. All forms of CG are hydrophilic and insoluble in organic solutions. The extent of sulfate and cations equilibrium, e.g., K^+^ and Na^+^ determine the water solubility, viscosity, and gel-formation capability of CG. Increasing the concertation of cross-linkers, or some key factors such as gums, salt, and organic compounds, can increase the viscosity of CG [13].

### 3.2. Agar and Agarose—Structural and Functional Entities

Agar is a polysaccharide consisting of agarose and agaropectin and predominantly found in genera of the Gracilariaceae and Gelidiaceae families [9]. Agarose (poly-3-*β*-d-galactopyranosyl-(1-4)-*α*-l-3,6-anhydrogalactopyranosyl-1) exists as a linear structure that is composed of repeating units of the agarobiose disaccharide (*β*-d-galactopyranosyl-(1-4)-α-l-3,6-anhydrogalactopyranose) [14]. The double-helical structure of agarose aggregates to develop a three-dimensional network that exhibits the water-holding capacity during elution. Two disaccharides namely d-galactose and 3, 6-anhydro-l-galactose units in repeated form constitute Agaropectin. It is more complicated in structure and acidic in nature due to the presence of pyruvic acid, sulfonic, and d-glucuronic acid residues, which discriminate agaropectin from the agarose. These residues might affect the gel-forming properties of agar [14]. Agar is soluble in boiling water, and its solution forms a resilient gel at about 32–40 °C, which can only be liquefied above 80 °C. Differences in melting and gelling temperatures of agar render agar polysaccharide a prevalent lubricant, emulsifier, and thickener in food, microbiological and pharmaceutical sectors [15].

### 3.3. Alginate—Structural and Functional Entities

Alginate is a linear polysaccharide composed of two building blocks including *α*-l-guluronic acid (G) and *β*-d-mannuronic acid (M) residues [16]. The blocks consist of either successive M residues (MMMMMM), G residues (GGGGGG), or alternating G and M residues (GMGMGM) [17]. The contents of the M and G blocks, as well as the length of each block, vary and depend on the seaweed source used to extract the alginate [18]. For instance, commercial products comprise only 14%–31% G blocks, whereas the alginate obtained from *Laminaria hyperborea* contains 60% G blocks [16]. Alginates are mainly extracted from brown seaweeds such as *Ascophyllum*, *Durvillaea*, *Lessonia*, *Ecklonia*, *Laminaria*, *Macrocystis, Turbinaria*, and *Sargassum* species either in sodium or calcium forms [19]. The salt form of alginic acid (for instance, Na-alginate) is the main constituent of the brown seaweeds cell wall constituting 17% to 47% of the total dry weight of the algae [20]. Alginate is a bioactive, biocompatible, non-toxic and biodegradable polymer and possesses stabilizing, thickening, and gel-forming attributes [20]. Initially, synthesized alginate entirely comprises poly-M residues that are subsequently epimerized to guluronic acid by the action of a mannuronic acid-epimerase enzyme. Biophysical and biochemical properties of alginate are reliant on the characteristic G:M ratios and molecular weights. G blocks are thought to exert an influential role in the binding of alginic acid with Ca^++^ and H^+^ ions and, thus its gelation [21]. Alginate is essentially not soluble in aqueous solutions. Interactions of monovalent ions such as ammonium and sodium with the carboxyl groups of alginic acid form water-soluble colorless and translucent salts with a diverse viscosity range. The use of some additives such as glycerol and dextran markedly alter the viscosity of alginic acid solution. The gelatin of alginate can be induced by divalent alkali metal ions (such as Mg^+2^, Ca^+2^, Ba^+2^, and Sr^+2^) without any cooling or heating conditions [22].

### 3.4. Fucoidan—Structural and Functional Entities

**Figure 4:**
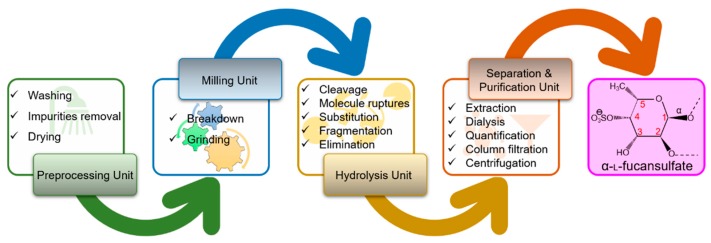
Mechanistic illustration of processing steps involved in the extraction of fucoidan (*α*-l-fucansulfate).

Fucoidans designate a type of polysaccharide that consists of significant quantities of l-fucose and sulfate ester groups accompanied with slight percentages of other monosaccharides, i.e., arabinose, xylose, glucose, galactose, and mannose. This polysaccharide constitutes one of the predominant components of the cell wall of brown algae and is thought to demonstrate protecting functions in desiccation stress that occurs under the low tide environments [23]. They have been reported to have been extracted from different seaweed sources such as *Ascophyllum nodusum*, *Ecklonia cava*, and *Undaria pinnatifida* [24]. Though fucoidan explicitly makes only 10%–20% of the total dry weight with the maximal documented content being 20% in *Fucus vesiculosus* [23]; its content widely fluctuates depending on different species of the algae and seasonal variations. Figure 4 shows the processing steps involved in the extraction of fucoidan.

### 3.5. Laminaran—Structural and Functional Entities

Laminaran is a principal storage glucan in all brown seaweeds with *Saccharina* and *Laminaria* species exhibiting the higher contents. However, it is also present in lesser quantities in other seaweed species such as *Fucus*, *Ascophyllum*, and *Undaria*. Although laminaran polysaccharide differs depending on habitat and seasonal variations, its content can reach up to 32% of the total mass of brown seaweeds [25]. Structurally, the linear structure of laminaran is made up of a *β*(1,3)-glucose in the backbone molecule that radiates outwards like branching at *β*(1,6). This *β*-glucan possesses a molecular mass ranging from 2.9 to 3.3 kDa covering between 50%–69% of d-glucose along with about 1.3% d-mannitol [9]. Among different algae, the proportion *β*(1,3)-glucose to *β*(1,6)-glucose substantially varies, and their molecular mass also depends on polymerization degree. The molecular weight of laminaran is notably affected by the extraction duration and solvent used, where an extended extraction protocol results in greater molecular weight [26]. As an example, *Saccharina longicruris* derived laminaran generally range from 2.89 to 3.32 kDa. In comparison to H_2_SO_4_, the use of HCl results in the extraction of higher molecular weight laminaran from *Saccharina* and *Laminaria* species [26]. In addition, environmental parameters also influence the structural and functional properties of laminaran [27].

### 3.6. Ulvan—Structural and Functional Entities

Ulvan is a sulfated and highly charged polyelectrolyte composed of recurrent disaccharides units (xylose and rhamnose) and glucuronic acid [12]. This peculiar configuration illustrates a novel source of functional biopolymers, and some rare sugars, viz., iduronic acid, and sulfated rhamnose can be derived from this unusual structure, which subsequently could be converted to a wide range of valuable compounds [28]. For example, sulfated polyaldobiuronan can be exploited as precursors to produce aromatic chemicals [28], whereas heparin equivalents with potential antithrombotic activities are synthesized from a rare sugar iduronic acid [29]. Thus, numerous synthesis steps are circumvented by the extraction of glucuronic acid from a natural resource [30]. Ulvan is extracted with hot water, the same as many other polysaccharides. Water-soluble and insoluble cellulose-like material are the two main categories of ulvans that have been recognized. Ulvans exist with an average molecular mass ranging from 189 to 8200 kDa. The presence of Ca^2+^-sequestering agents, alkali or acid in the water caused a substantial improvement in its yield. Ulvan with enhanced purity level is achieved by ethanol precipitation followed by freeze-drying concentration. Nevertheless, the specific composition and yield of ulvan can be affected by environmental factors, the type of seaweed, the collection season and the extraction procedure adopted [31].

### 3.7. Furcellaran—Structural and Functional Entities

Furcellaran is produced by the red seaweed, *Furcellaria fastigiata*. For furcellaran extraction, the dry seaweed is treated by alkali and extracted with hot water and vacuum concentration. The isolated gel threads are freeze concentrated, and then centrifuged or pressed to eliminate the excess water. The structural arrangement of furcellaran is composed of d-galactose, 3, 6-anhydro-d-galactose and sulfated contents of both sugars. Structurally, furcellaran very much resembles k-carrageenan; however, the k-carrageenan possesses relatively lower sulfate groups (1% to 5%), whereas furcelleran comprises between 16% to 20% ester sulfates [32]. The concentration of furcelleran needed for gelation is ranged from 0.2%–0.5% with varying sugar contents. Porphyran is a sulfated polysaccharide constituting up to 48% of the dry weight of the seaweed belonging to *Porphyra s*pp. It resembles agar chemically with a linear backbone of alternating *β*-d-galactose and 3,6-anhydro-*α*-l-galactose residues. It has shown apoptotic, antioxidant and antihyperlipidemic activities [15].

## 4. Biomedical Values of Engineered Cues

### 4.1. Targeted Drug Delivery

The application of natural polymer-based NPs has appeared a remarkable approach for targeted delivery of drugs owing to the benefits provided by their small sizes. Grenha et al. [33] fabricated carrageenan and chitosan (marine-derived natural biopolymers) blended novel nanoparticles to investigate their efficiency as carriers for meticulous release of therapeutic macromolecules. The nanoparticles were formulated under very gentle conditions and hydrophilic environments, thus circumventing the employment of toxic organic solvents or other unfriendly techniques for their synthesis. The as-prepared nano-carriers (dimensions ranged from 350 to 650 nm) revealed exceptional capacity for the controlled release of a model protein ovalbumin for up to 3 weeks with an elevated loading capacity of 4%–17%. Moreover, the nanoparticles exhibited high biocompatibility representing no cytotoxicity in bioassays performed against L929 fibroblasts. A series of oxidized Na-alginate (ALG) and hyaluronic acid-functionalized with hydrazide and thiol (HA) based hydrogels (HA/ALG) were fabricated via disulfide and hydrazone bonds and investigated their drug-releasing performance using bovine serum albumin (BSA) as a model drug. Results illustrated an in-vitro cumulative BSA release of almost 79%, 72% and 69% by HA2/ALG2, HA3/ALG3, and HA4/ALG4 hydrogels after 20 days [34]. An emulsion cross-linking approach was adopted to design hydroxyapatite/chitosan/sodium alginate composite beads with potential biocompatibility in the presence of Ca^2+^-ions as a cross-linker. The encapsulation and drug-loading efficiency of these composite microspheres were much greater as compared to HA NPs. Doxycycline-incorporated hydroxyapatite/chitosan/sodium alginate composites showed an effective pH-responsive release of drugs and found to be cell and blood compatible. Moreover, the newly synthesized microspheres presented superior cell adhesive and propagation capability compared with HA-based NPs and HA/SA bio-composite microbeads [35].

Oligocarrageenan was synthesized by an enzymatic degradation process using *κ*-carrageenan extracted from red seaweed *K. alvarezii* and modified with polycaprolactone (PCL) chains adopting a protection/deprotection technique. Hydrophobic drugs i.e., curcumin was effectively loaded onto the resultant PCL-grafted oligo-carrageenan copolymers (187 nm size) and released within 24 to 72 h. The copolymers were observed to be non-cytotoxic and stimulated the curcumin uptake by endothelial EA-hy926 cell lines. The anti-inflammatory activity of curcumin was increased in tumor necrosis factor-triggered inflammatory trials. Lastly, no toxicity was recorded in zebrafish that substantiated the exploitation of oligo-carrageenan as nano-carriers for delivering hydrophobic molecules to various organs, such as the brain, lung, and liver [36]. For the first time, Pozharitskaya et al. [37] presented pharmacokinetic and tissue distribution of fucoidan obtained from *Fucus vesiculosus* (brown seaweeds) after oral administration to rats. This study also signifies the importance of pharmacokinetic profiling of active compounds, which is essential for drug development and approval.

### 4.2. Wound-Curative Potential

Treatment of wound infection is a key research domain necessitating considerable attention to deal with such pathogenic conditions. Wound repair or healing is a highly coordinated biological process that requires various forms of cell rejuvenation, such as collagenation, epithelization, and tissue remodeling [38,39]. Several advanced research-based studies have directed their focus to explore new biologically active compounds with remarkable tissue repairing and scar formation-limiting abilities. Polysaccharides extracted from seaweeds have gained a particular interest in the tissue engineering domain owing to their abundant availability, non-toxicity, low-cost, biodegradability, and bio-renewable characteristics. In these scenarios, extensive work has been executed on seaweed polysaccharide-derived novel composite materials for wound healing and tissue regenerative properties. O’Leary et al. [40] reported that fucoidan modulates the effect of transforming growth factor (TGF)-*β*1 on fibroblast proliferation and wound repopulation in in vitro models of dermal wound repair. The study reports the interaction between two commercial preparations of fucoidan and TGF-*β*1. As-prepared fucoidan and heparin based preparations were able to inhibit fibroblast proliferation at concentrations from 0.01 to 100 mg/mL. Feki et al. [41] evaluated the potential wound curative properties of *Falkenbergia rufolanosa* polysaccharide (FRP) strengthened by polyvinyl alcohol (PVA). The hydrogel films were composed of various proportions of PVA (100%), F1 (FRP/PVA, 70:30), F2 (FRP/PVA, 50:50), and F3 (FRP/PVA, 30:70). Among the different combinations, F1 (FRP/PVA, 70:30) significantly promoted the healing of induced wound after 8 days of treatment evinced by greater collagen content relative to the control, and PVA-treated groups. In vivo wound curative effects of a novel sorghum polysaccharide extracted from the seeds of *Sorghum bicolor* was assessed to treat fractional CO_2_ laser-induced burns. Results demonstrated that the use of sorghum polysaccharide-based hydrogel on the burn microenvironment meaningfully improved the appearance of the wound and hastened wound cessation after eight days of treatment in a rat model. Moreover, the resultant hydrogel showed no hemolytic effect on human red blood cells. A complete re-epithelialization of injuries with an entire epidermal renewal evidenced by histological examination of injured tissues manifest sorghum polysaccharide-based hydrogel as an efficient wound-restorative candidate in contemporary medicine [42].

In recent times, the utilization of natural/synthetic polymer-based gel materials, composites, films, micro- and nano-particulate systems has been escalated to develop bioinspired materials, especially for the management of wounds. Janarthanan and Senthil Kumar [43] investigated the application of textile fabrics modified with sodium alginate extracted from two different seaweed sources including *Padina tetrastromatica* and S*argassum wightii*. Experimental results that the *S. wightii* based textile fabric displayed superior wound curative potentiality in contrast to a textile fabric coated with alginate isolated from *P. tetrastromatica* and could be employed as preferable materials for skin grafting, wound healing, hygienic textiles, and pharmaceutical industries. Laminaran, obtained from marine algae presents interesting features of biodegradability, non-toxicity, and hydrophilicity. The efficacy of *Cystoseira barbata* laminaran (a brown seaweed)-based cream was evaluated for curing full-thickness wounds induced in rats. It was observed that the formulated cream comprising laminaran as a bioactive constituent exerted significant wound-improving effects, and after 13 days, the wound reduction reached 98.57%. As compared with the untreated groups, the derma in laminaran-treated group was correctly organized revealing increased fibroblast densities as well as enhanced deposition of collagen [44]. Very recently, fucoidan, from brown algae, has been used for transdermal formulations targeting inflammatory skin conditions or as an anticoagulant after topical application, which is a very important aspect of wound healing [39,45]. Obluchinsksya et al. [45] investigated the effects of ultrasound treatment on the chemical composition and anticoagulant properties of dry extract of bladderwrack (*Fucus vesiculosus*). As-developed dry extract of bladderwrack displayed higher anticoagulant upon local administration to Wistar rats.

### 4.3. Anticancer Potentialities

**Figure 5:**
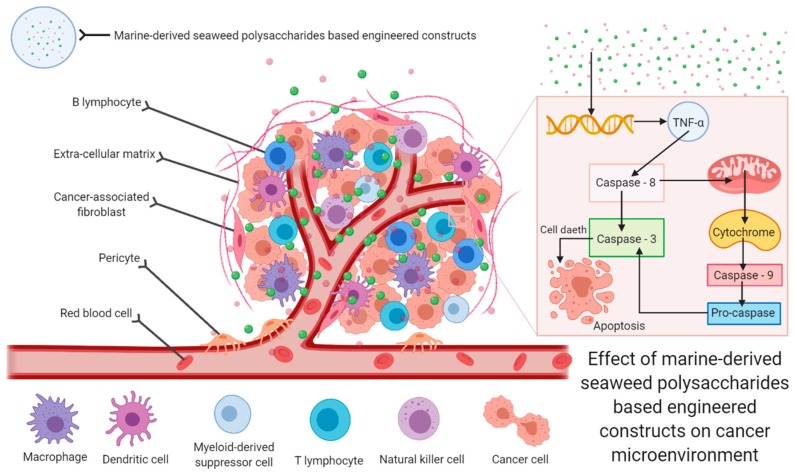
Anticancer potentialities of marine-derived seaweed polysaccharides based engineered cues to destroy cancer cells and prevent metastasis by acting on tumor cells microenvironment via different mechanisms such as cell death via apoptosis.

Cancer is one of the leading diseases detected in developed countries with the appearance of approximately 1.6 million new cases in 2017 in the USA alone. Currently available drugs for cancer treatment, including taxol, doxorubicin, cyclophosphamide, and 5-fluorouracil are invariably associated with numerous adverse side effects such as anemia, alopecia, fatigue, immunosuppression, neurological issues, peripheral neuropathy, and fertility problems. Polysaccharides extracted from marine sources have arisen as potentially effective chemical entities that have shown significant anticancer activities against a wide range of cancer cells. Over 100 different polysaccharides have been studied in the last decade, and many of them have exhibited the potential to address the shortcomings of traditional chemotherapeutic treatments. They are reported to destroy cancer cells and prevent metastasis by acting on tumor cells via different mechanisms such as cell cycle arrest, DNA damage, nitric oxide production, and depolarization of mitochondrial membrane (Figure 5).

Chen et al. [46] examined the extraction, characterization, and anticancer and immune-stimulation activities of sulfated heteropolysaccharide from *Tribonema* sp. (TSP) against HepG2 and RAW264.7 macrophage cell lines. Experimental results revealed a substantial immunomodulatory activity of the sulfated polysaccharide by upregulating TNF-*α*, and interleukin 6 and 10. An MTT bioassay determined a potential anticancer activity towards HepG2 cells that was mainly attributed to induce cellular apoptosis than that to arrest the mitosis and cell cycle. These findings suggest that TSP might have potential as an anticancer resource. Different methods were used to extract fucoidan, a sulfated polysaccharide, from the brown seaweed *Nizamuddinia zanardinii* and appraised its molecular properties, immune-promoting and anticancer activities. Notably, fucoidans presented an appreciable anticancer activity that ranged from 21.31%–55.94% and 26.77%–67.46% for HepG2 and HeLa cells, respectively. In addition, polysaccharides isolated using all methods considerably induced macrophage cell lines to release nitric oxide, and Alcalase-assisted extracted polysaccharide exerted the most pronounced immune-enhancing activity [47]. In a recent study, Prabhu and coworkers, [48] assessed the anticancer potential of methyl gallate-incorporated zeolitic imidazole framework (MG@ZIF-L) against lung cancer cell lines A549 synthesized by using *Gracilaria debilis* (red seaweed) and. Results demonstrated that the newly synthesized nanoplatform showed biocompatibility, high loading capacity, and rapid release of tested drugs in the cancer microenvironment. In vitro antitumor trials confirmed the high cytotoxic potentialities of MG@ZIF-L against A549 cells. The level of intracellular reactive oxygen species (ROS) causing mitochondrial impairment was enhanced by MG@ZIF-L as illustrated by Fluorescent microscopy. In addition, in vivo and in vitro toxicity evaluation using zebrafish embryo and peripheral blood mononuclear cell revealed the nontoxicity of methyl gallate-based nanohybrid composite material, indicating its promise as a versatile biocompatible agent for lung cancer remedy.

**Figure 6:**
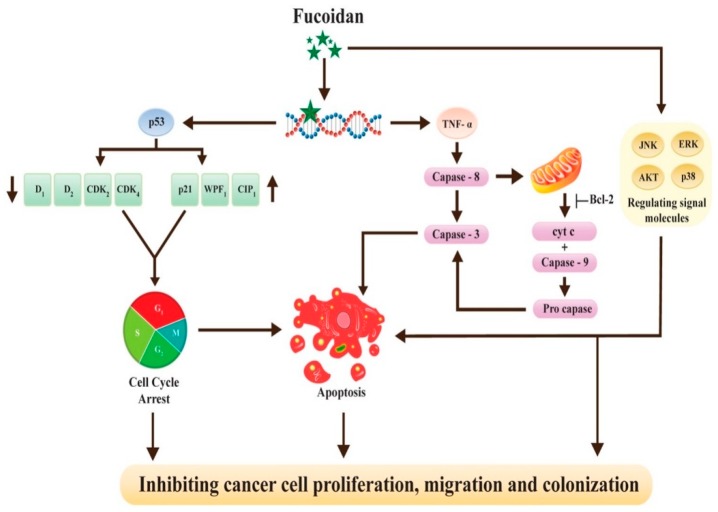
Anticancer activity of brown seaweed (*Turbinaria conoides*)-derived fucoidan in HepG2 cancer cells. Reprinted from Arumugam et al. [54] with permission under the CC BY-NC-ND license (http://creativecommons.org/licenses/BY-NC-ND/4.0/).

Many practical in vitro reports have demonstrated the functionality of fucoidan against various cancers such as melanoma and hepatocarcinoma [49]. Although the molecular mode of action of fucoidan has not been explicitly elucidated, some recent reports described that the anticancer effects rely on activation through apoptosis and inhibition of angiogenesis and metastasis in various types of cancer cell lines. Notably, the anticancer potentialities of fucoidan polysaccharide have been precisely reported in breast, lung, colon, prostate, bladder and liver cells [50,51]. A recent study conducted using the MCF-7 breast cancer cells demonstrated the promiscuity of fucoidan as a suitable candidate for the treatment of cancer in amalgamation with doxorubicin, cisplatin, and taxol drugs [52]. Furthermore, the cancer tumor-suppressing capability of fucoidan increased the overall survival rate in patients associated with cancer [53]. Recently, Arumugam and coworkers, [54] meticulously investigated the anti-cancer properties of brown seaweed extracted sulfated polysaccharide, fucoidan in a hepatoblastoma-derived (HepG2) cell line by an array of advanced techniques such as colony formation, cell viability, cell cycle progression, cell migration, apoptosis and genetic damage accompanied by mitochondrial membrane and nuclear morphological potential. It was found that cells presented appreciable cell viability when constituted with different fucoidan/quercetin standards. In contrast, as control, fucoidan-constituted cells profoundly congregated proliferative cells in the G0/G1 stage of the cell cycle. DNA damage was remarkably enhanced by the steadily increasing level of fucoidan from 50 to 200 μg/mL. Conclusively, the fucoidan presented an encouraging anticancer potentiality against HepG2 cancer cell lines by suppressing cell propagation, and cell detention, which was interrelated with apoptosis and genetic damage (Figure 6) [54]. Vaikundamoorthy et al. [55] explored *S. wightii* polysaccharides as a new and renewable source of naturally occurring anticancer compounds. They carried out the isolation and purification of two polysaccharide fractions namely SWP1 and SWP2 from the crude extract of brown seaweed *S. wightii* for their anticancer activity. The anticancer profile revealed that the purified polysaccharides, in a dose-dependent mode, profoundly reduced the propagation of two kinds of breast cancer cell lines i.e., MCF7 and MDA-MB-231. Furthermore, they led to apoptosis of the tested cell lines by enhancing the generation of ROS, increasing the activity of caspase 3/9, nuclei impairment and cleavage of the mitochondrial membrane.

### 4.4. Tissue-Engineering Aspects

**Figure 7:**
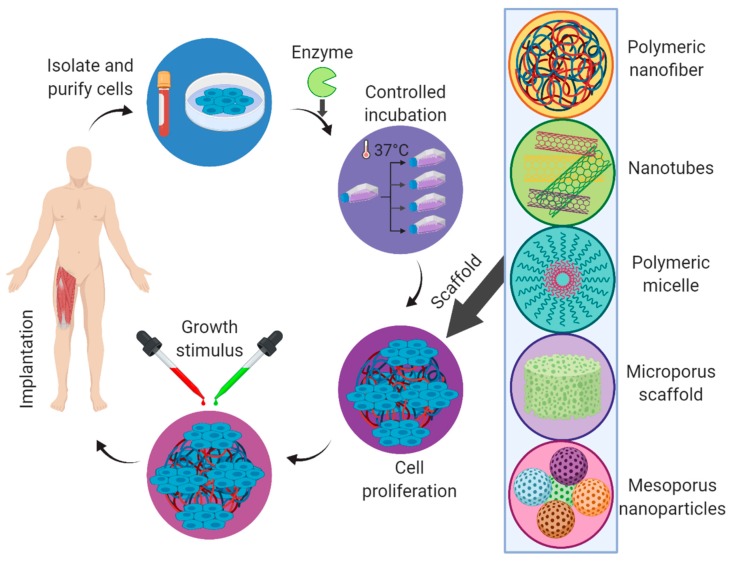
Various processes involved in the tissue engineering to develop a tissue-engineered scaffold for implantation purposes.

Tissue engineering is a multidisciplinary field of research and development including cell biology, biotechnology, and materials science and engineering for repairing injured or malfunctioning tissues or organs as a substitute to whole organ transplantation (Figure 7) [56,57]. Incredible progress has been dedicated in the last decade to the field of tissue engineering for an array of therapeutic applications. The ultimate objective of this research is to design potential biological alternatives for implanting into the body or to foster tissues remodeling in a frame of a three-dimensional scaffold. Definite requisites for the development of ideal natural polymer-derived bioengineered scaffolds include the high porosity with huge specific surface area, appropriate distribution of pore size, designable surface chemistry sufficient structural integrity, and controllable biodegradability with degradation rate closely matching the rate of new tissue formation. Moreover, the engineered scaffold should be biocompatible, non-toxic, and have the ability to positively interact with cells to stimulate cell adhesion, migration, proliferation, and distinguished cell functionalities [58,59].

Fucoidan helps in mineral deposition in the bone matrix by increasing the expression of type-I collagen, osteocalcin, and the activity of alkaline phosphatase enzyme [60]. Recent studies have demonstrated the biological activity of fucoidan in the treatment of osteoarthritis. Oral administration of fucoidan extracted from *Undaria pinnatifida* effectively relieved pain in an animal model of collagen-triggered arthritis [61]. Over 50% symptoms of osteoarthritis were reduced by the ingestion of fucoidan-enriched seaweed extracts [62]. A substitute to commercial bone was designed by using low molecular weight fucoidan having to foster the bone tissue-regeneration capability [63]. The authors determined that fucoidan treatment helped in promoting human osteoblastic proliferation, and type-I collagen expression which, in turn, enhanced ALP activity with efficient mineralization of bone tissue. Polycaprolactone-fucoidan containing biocomposites showed excellent bone mineralization and cellular proliferation for bone reinforcement. Such biocomposites were reported to provide improved biological stimulation for the regeneration of bone constructs [64,65]. In another study, the mineral deposition was enhanced to 30% in a composite scaffold incorporated with fucoidan polysaccharide [63].

Alginate is another well-recognized scaffolding material used to reconstruct lost bone and treat various organ defects. Alginate-based biocompatible hydrogels have been applied for the regeneration of bone and cartilage, as well as stem-cell transplantation [66]. Nonetheless, there has been inadequate examination of the mechanical strength for utilization in amalgamation with some other suitable polymer to improve mechanical integrity for bone regeneration [67]. Fabrication of chitosan-alginate gel/bone morphogenetic protein-2/mesenchymal stem cells blended bio-composites has presented interesting properties for exploitation as an injectable biomaterial for new bone tissue regeneration [68]. Alginate/chitosan hybrid structures led to improve the mechanical strength and structural stability, as well as accelerated vascularization and promoted osteogenesis following embedment in the bone [69]. A porous three-dimensional matrix composed of calcium phosphate cement in combination with alginate has been employed as a novel scaffold for bone-tissue engineering and drug-delivery applications [70]. Fabrication of alginate, chitosan, collagen and hydroxyapatite-based composite with better porosity were found to be interesting candidates for bone tissue regeneration [71]. Alginates-based gel modified with proline-histidine-serine-arginine-asparagine with different sequences and ratios could be fascinating scaffolds for a wide range of bone-tissue engineering purposes [72]. The suitability of two different polymer scaffolds, such as chitosan-alginate (Chi-Alg) and fucoidan containing chitosan-alginate (Chi-Alg-fucoidan) was evaluated as a substitute for bone implantation. In vitro experimental trials showed greater cell proliferation, promising compatibility towards MG-63, and increased secretion of alkaline phosphatase using the Chi-Alg-fucoidan biocomposite than a Chi-Alg polymer scaffold. As compared to the Chi-Alg scaffold, the mineralization and adsorption of protein were two times higher in the case of Chi-Alg-fucoidan biomaterial that indicates its applicability for the regeneration of bone tissue [73].

The proliferation of chondrocytes was promoted using scaffold fabricated by combining alginate, gelatin and 2-hydroxyethyl methacrylate. Importantly, high glycosaminoglycan and collagen contents were determined in the cells by applying alginate incorporated scaffold [74]. Ayoub et al. [75] anticipated that the incorporation of laminaran polysaccharide from a brown seaweed *Saccharina longicruris* could drive up the matrix deposition and thus accelerate the tissue-generation process. They allowed producing a dermis by culturing monolayer human skin fibroblasts in the presence of seaweed-derived laminaran for 7 or 35 days. It was found that laminaran treatment did not cause any alterations in the content of smooth muscle actin or the growth efficacy, but the collagen-I deposition was substantially increased in a concentration-mediated route. After cultivation for 35 days, the new assembled dermal thickness, and collagen-I deposition were meaningfully increased in the presence of laminaran. Bhadja et al. [76] investigated the repairing effects of six seaweed polysaccharides (low molecular weight) including *Eucheuma gelatinae* polysaccharide, *Laminaria japonica* polysaccharide, and degraded *Gracilaria lemaneiformis*, *Porphyra yezoensis*, *Undaria pinnatifida* and *Sargassum fusiforme* polysaccharides on oxalate-triggered injured human kidney epithelial cells (HK-2) by cell morphology and viability bioassays. Experimental results demonstrated that all the tested polysaccharides did not exert any cytotoxic effect on HK-2 cells, and exhibited notable repair activity on damaged HK-2 cell lines. Important to mention is the better repair effect of degraded polysaccharides on damaged cells as compared to their native counterparts.

Extracellular polymeric substances (EPS) originating from brown seaweeds are believed to have bioactivities such as antioxidant and tissue engineering properties. Primary rat astrocytes were cultivated on EPS incorporated electrospun polycaprolactone (PCL) nanofibrous mat. Two major components of EPS including fucoidan and laminarin were determined to modulate astrocyte activities in a concentration-dependent manner. The viability of astrocytes tends to increase at an EPS concentration below 10 μg/mL. In contrary, addition of higher than 10 μg/mL of EPS in media inhibited the astrocytes viability. Results indicate that EPS-based PCL nanofibers could be used as a nanoscaffold for the treatment of injuries in central nervous system [77]. Popa and coworkers [78] discussed the usefulness of kappa-carrageenan-based hydrogels in the remedy of cartilage tissue damages. The hydrogels were fabricated by adopting a gelation method by encapsulating human adipose stem cells (hASCs) in kappa-carrageenan solution (1.5% *w/v*), and the resulting cell-encapsulated hydrogels were allowed to culture for 21 days. Results evidenced the supporting role of k-carrageenan hydrogels in proliferation, viability, and chondrogenic differentiation of tested human stem cells.

### 4.5. Ultraviolet (UV) Protectant Potential

**Figure 8:**
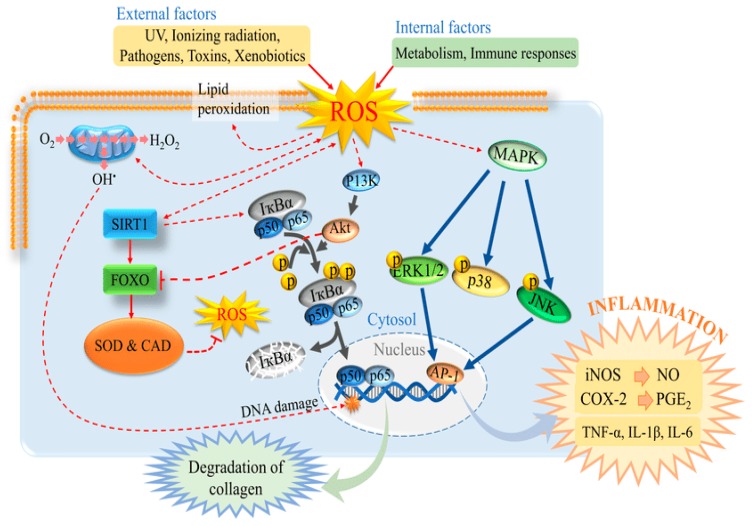
Reactive oxygen species (ROS)-induced tissue damage. Exposure of skin cells to various exogenous factors triggers a series of signaling pathways that lead to inflammatory responses and induce the degradation of connective tissues. Reprinted from Priyan Shanura Fernando et al. [84] with permission from Taylor & Francis. Copyright (2019) Informa UK Limited, trading as Taylor & Francis Group.

Extrinsic skin aging is induced by exposure to different kinds of UV radiation, i.e., UV-A (320–400 nm), UV-B (290–320 nm), and UV-C (100–290 nm) [79]. Among these, UV-A and UV-B have the ability to enter the atmosphere resulting in damaging effects to humans. For instance, exposure to UV-B is 1000-times more detrimental, cytotoxic and mutagenic than that to UV-A [80]. Besides, UV-B plays a key role in the progression of photoaging as it reduces type-I procollagen levels and increases matrix metalloproteinases-1 (MMP-1) levels in human skin [81]. Excessive exposure to UV irradiation leads to changes in the skin with the manifestations of wrinkles, increased degradation of collagen, dryness, coarseness, epidermal thickness, spotted skin-coloration, etc. [82]. In addition, UV irradiation stimulates ROS generation, which induces inflammatory response resulting in oxidative damage to dermal protein [83]. Figure 8 portrays the skin-damaging effects of ROS and the initiating pathways leading to disorganization and inflammation of connective tissues [84]. ROS explicitly has shown to promoted TNF and interleukins secretion, which interacts with some critical proteins in nuclear transcription factor kappa *β* and mitogen-induced protein kinase pathways leading to collagen degradation [85].

Seaweeds-derived bioactive polysaccharides have garnered exceptional interest owing to their anti-photoaging, anti-inflammatory, antioxidant, and antitumor bioactivities. A bio-active compound, Sargachromanol E, extracted from the brown alga *Sargassum horneri* has increased scavenging ability against ROS and prevented cell membranes from oxidative modification in UV-exposed human dermal fibroblasts [86]. Sargachromanol E treatment-maintained skin collagen fibers by inhibiting the expression of metalloproteinases (collagen-degrading matrix). In addition to antioxidant potentiality, *S. horneri*-derived polysaccharides exhibited potent moisture-absorption and maintenance capabilities [87]. Li et al. [88] evaluated the usefulness of *S. glaucescens* extracts in skincare by carrying gout in-vitro bioassays in epidermal keratinocytes and dermal fibroblasts. A 2,2-diphenyl-1-picrylhydrazyl (DPPH) scavenging assay confirmed the antioxidant activity of extract by inhibiting the production of H_2_O_2_-induced ROS in dermal fibroblasts. In contrast to the control, the application of extracted alga extract treatment showed a 2.95-fold increased wound healing of CCD-966SK fibroblasts along with improved cell regeneration following UV-A irradiation. At the molecular level, extract treatment led to increased expressions of DNA repair regulatory genes (*XRCC1* and *ERCC6*), as well as antioxidant genes (*GPX1* and *SOD1*) after UV-A light exposure that indicated its favorable impacts on dermal cell protection and cell regeneration against UV light. Application of *S. glaucescens* extract also augmented the propagation of epidermal keratinocytes and provoked the expression of genes related to the skin barrier (*TGM1*, *KRT14,* and *KRT10*) in keratin-producing cells. Polysaccharides derived from *S. fusiforme* possesses promising free radical capturing, anti-peroxide and tumor inhibition, activities. *S. fusiforme* polysaccharide (SFP) has reported exhibiting substantial UV-B protecting activity in hairless Kun Ming rats by the manipulation of spleen and thymus indexes, and skin water content. Results showed that the extracted SFP alleviated UVB-triggered oxidative stress by increasing the catalytic activities of catalase and superoxide dismutase and diminishing the malondialdehyde and ROS levels. SFP treatment also suppressed the levels of MMP-1 and 9 [89]. It was found that the administration of different dose levels of SFP significantly reduced the thickness of both dermis and epidermis and attenuated the sebaceous hyperplasia. Moon et al. [90] investigated the inhibitory influence of *Costaria costata*-based fucoidan on the UV-B-induced matrix metalloproteinase-1 (MMP-1) promoter, mRNA, and protein expression. The immortalized human keratinocyte (HaCaT) cell line was used under in-vitro conditions. An earlier study by Fitton et al. [91] measures the effect of the fucoidan-rich extracts on enzyme inhibition, glycation, antioxidant activity and Sirtuin 1 (*SIRT1*) protein expression. Considerably, the tested extract of marine microalgae, i.e., *Undaria pinnatifida* aided the skin immunity which is an important element for skin protection from harmful damage.

## 5. Current Challenges and Literature Gaps

Although the current biotechnological and nanotechnological advancement has made a significant improvement in the medical sector of the modern world. However, several of the proposed methodological procedures and their resulting engineered constructs are still being conceptualized and facing challenges due to poor pharmacokinetics and inefficient or ineffective drug delivery. With particular reference to the use of marine-based seaweed polysaccharides to engineer cues with therapeutic and biomedical values are lacking standardized procedures. This has resulted in notable literature gaps, as at present, insufficient research and scientific literature efforts have been made towards the effective and sustainable exploitation of seaweed polysaccharide-based unique materials in the biomedical settings of the 21st-century, which still remains untapped to a great extent. Such inadequate exploitation, in turn, results a huge variation in the end product based on the same material. Other challenges include the short life span of drugs in the natural body environment, inefficient delivery, burst release, safer way out, and complications of treatment sites, such as tumor sites. In this context, considerable efforts have been made and most of them have been improved to a certain extent. However, for a successful deployment of materials-based therapeutic cues need further in-depth understanding are of supreme interest. Moreover, the development of prudent processing and methodological procedures for engineered cues with therapeutic and biomedical values could be a trending focus to present the potentialities of seaweed polysaccharides.

## 6. Concluding Remarks and Outlook

In conclusion, engineering therapeutic cues with unique structural and functional entities is an equally innovative and ground-breaking platform with an optimistic vision in numerous biomedical areas. To further strengthen the biomedical potentialities, marine-derived seaweed polysaccharide-based engineered constructs signify progressive properties with tremendous applications. This review presents numerous seaweed polysaccharides, such as carrageenan, ulvan, fucoidans, alginate, agar, laminaran, and furcellaran, as potential candidate materials to engineer novel cues with biomedical values to tackle the limitations of counterparts in practice. Most of these counterparts in practice have become ineffective or inefficient for 21st-century medical settings at large and for drug delivery, in particular. The challenges discussed above can be effectively tackled by deploying or imparting the inherited bioactive features of seaweed polysaccharides. Seaweed polysaccharides are biologically tunable, biocompatible, biodegradable, renewable, and of a non-toxic nature, which urges researchers to use them to design therapeutically effective, efficient, patient-compliant, and age-compliant drug delivery and tissue-engineered platforms. From the functional perspectives, based on their significant retention capabilities, tunable active units, swelling and colloidal features, marine-based seaweed polysaccharides have appeared as highly useful materials for modulating drug-delivery and tissue-engineering systems without compromising their inherited features.

From the perspective of wound-curative potential, an array of seaweed-based polysaccharides, along with other biologically active cues have been developed and exploited. As discussed above with suitable examples, the reported literature indicates state-of-the-art advances to treat the wounds of numerous categories and related skin disorders using seaweed-derived bioactive polysaccharides. For instance, fucoidans consisted of significant quantities of l-fucose and sulfate ester groups accompanied with small percentages of other monosaccharides i.e., arabinose, xylose, glucose, galactose, and mannose has been used as an anticoagulant, which is a very important aspect for effective wound healing. Moreover, fucoidans isolated from several marine sources displayed significant inhibitory activities against various cancer types. However, further in-depth research is needed to understand the exact action mechanism responsible for cancer cell inhibition by seaweed extracts or polysaccharides. Besides the well-reported anti-photoaging, anti-inflammatory, antioxidant, and antitumor bioactivities of seaweed-derived bioactive polysaccharides, the increased scavenging ability against ROS is of supreme interest with particular reference to UV-protectant features. Considering all these points, marine-based seaweed polysaccharides remain a subject of intensive future research.

## Figures and Tables

**Table 1:** Literature search results obtained from the PubMed database.

Search Terms	Total Articles	Number of Articles Published in the Last Five Years Filtered with Best Match Term on					
		2019	2018	2017	2016	2015	All Past Years
Marine seaweed polysaccharides	384	40	63	42	48	25	166
Drug delivery using polysaccharides	23,946	928	2030	1910	1880	1810	15,388
Wound healing using polysaccharides	6855	209	482	444	391	356	4973
Anticancer activities of polysaccharides	2280	170	272	256	232	233	1117
Polysaccharides based engineered carriers	1114	94	173	166	120	105	456

## Supplementary Materials

The following are available online at https://www.mdpi.com/1660-3397/18/1/7/s1, Table S1: Literature search results obtained from the Scopus database. The distribution of articles in each search category is based on total number of articles and reads from top to bottom column-wise.

## References

[1] Tiwari G., Tiwari R., Sriwastawa B., Bhati L., Pandey S., Pandey P., Bannerjee S.K. (2012). Drug delivery systems: An updated review. Int. J. Pharm. Investig..

[2] Bilal M., Rasheed T., Ahmed I., Iqbal H.M.N. (2017). High-value compounds from microalgae with industrial exploitability—A review. Front. Biosci. Sch..

[3] Sosa-Hernández J.E., Escobedo-Avellaneda Z., Iqbal H., Welti-Chanes J. (2018). State-of-the-art extraction methodologies for bioactive compounds from algal biome to meet bio-economy challenges and opportunities. Molecules.

[4] Albinali K.E., Zagho M.M., Deng Y., Elzatahry A.A. (2019). A perspective on magnetic core–shell carriers for responsive and targeted drug delivery systems. Int. J. Nanomed..

[5] Rasheed T., Nabeel F., Raza A., Bilal M., Iqbal H.M.N. (2019). Biomimetic nanostructures/cues as drug delivery systems: A review. Mater. Today Chem..

[6] Raza A., Rasheed T., Nabeel F., Hayat U., Bilal M., Iqbal H. (2019). Endogenous and exogenous stimuli-responsive drug delivery systems for programmed site-specific release. Molecules.

[7] Du Y., Chen B. (2019). Combination of drugs and carriers in drug delivery technology and its development. Drug Des. Dev. Ther..

[8] Craigie J.S., Cole K.M., Sheath R.G. (1990). Cell walls. Biology of the Red Algae.

[9] Kraan S., Chang C.F. (2012). Algal polysaccharides, novel applications and outlook. Carbohydrates—Comprehensive Studies on Glycobiology and Glycotechnology.

[10] Iqbal H.M., Rasheed T., Bilal M. (2018). Design and processing aspects of polymer and composite materials. Green Sustain. Adv. Mater. Process. Charact..

[11] Cardozo K.H., Guaratini T., Barros M.P., Falc˜ao V.R., Tonon A.P., Lopes N.P., Pinto E. (2007). Metabolites from algae with economical impact. Comp. Biochem. Physiol. Part C Toxicol. Pharmacol..

[12] Cunha L., Grenha A. (2016). Sulfated seaweed polysaccharides as multifunctional materials in drug delivery applications. Mar. Drugs.

[13] Campo V.L., Kawano D.F., da Silva D.B., Carvalho I. (2009). Carrageenans: Biological properties, chemical modifications and structural analysis—A review. Carbohydr. Polym..

[14] Synytsya A., Čopíková J., Kim W.J., Park Y.I., Kim S.K. (2015). Cell wall polysaccharides of marine algae. Springer Handbook of Marine Biotechnology.

[15] Venugopal V. (2019). Sulfated and Non-Sulfated Polysaccharides from Seaweeds and their Uses: An Overview. EC Nutr..

[16] Venkatesan J., Bhatnagar I., Manivasagan P., Kang K.H., Kim S.K. (2015). Alginate composites for bone tissue engineering: A review. Int. J. Biol. Macromol..

[17] Lee K.Y., Mooney D.J. (2012). Alginate: Properties and biomedical applications. Prog. Polym. Sci..

[18] Rehm B.H. (2009). Alginate production: Precursor biosynthesis, polymerization and secretion. Alginates: Biology and Applications.

[19] Rupérez P., Gómez-Ordóñez E., Jiménez-Escrig A. (2013). Biological activity of algal sulfated and nonsulfated polysaccharides. Bioact. Compd. Mar. Foods Plant Anim. Sources.

[20] Zia K.M., Zia F., Zuber M., Rehman S., Ahmad M.N. (2015). Alginate-based polyurethanes: A review of recent advances and perspective. Int. J. Biol. Macromol..

[21] Smidsrod O., Draget K.I. (1999). Chemistry and physical properties of alginates. Carbohydrate.

[22] Clementi F., Crudele M.A., Parente E., Mancini M., Moresi M. (1999). Production and characterisation of alginate from Azotobacter vinelandii. J. Sci. Food Agric..

[23] Berteau O., Mulloy B. (2003). Sulfated fucans, fresh perspectives: Structures, functions, and biological properties of sulfated fucans and an overview of enzymes active toward this class of polysaccharide. Glycobiology.

[24] Athukorala Y., Jung W.K., Vasanthan T., Jeon Y.J. (2006). An anticoagulative polysaccharide from an enzymatic hydrolysate of *Ecklonia cava*. Carbohydr. Polym..

[25] Bixler H.J., Porse H. (2011). A decade of change in the seaweed hydrocolloids industry. J. Appl. Phycol..

[26] Kadam S.U., Tiwari B.K., O’donnell C.P. (2015). Extraction, structure and biofunctional activities of laminarin from brown algae. Int. J. Food Sci. Technol..

[27] Rioux L.E., Turgeon S.L., Beaulieu M. (2007). Characterization of polysaccharides extracted from brown seaweeds. Carbohydr. Polym..

[28] Lahaye M., Robic A. (2007). Structure and functional properties of ulvan, a polysaccharide from green seaweeds. Biomacromolecules.

[29] Barsanti L., Gualtieri P. (2014). Algae: Anatomy, Biochemistry, and Biotechnology.

[30] Hinou H., Kurosawa H., Matsuoka K., Terunuma D., Kuzuhara H. (1999). Novel synthesis of L-iduronic acid using trehalose as the disaccharidic starting material. Tetrahedron Lett..

[31] Hernández-Garibay E., Zertuche-González J.A., Pacheco-Ruíz I. (2011). Isolation and chemical characterization of algal polysaccharides from the green seaweed Ulva clathrata (Roth) C. Agardh. J. Appl. Phycol..

[32] Imeson S.P., Phillips G.O., Williams P.A. (2010). Carrageenan and furcelleran. Handbook of Hydrocolloids.

[33] Grenha A., Gomes M.E., Rodrigues M., Santo V.E., Mano J.F., Neves N.M., Reis R.L. (2010). Development of new chitosan/carrageenan nanoparticles for drug delivery applications. J. Biomed. Mater. Res. Part A.

[34] Zhang Y., Li X., Zhong N., Huang Y., He K., Ye X. (2019). Injectable in situ dual-crosslinking hyaluronic acid and sodium alginate based hydrogels for drug release. J. Biomater. Sci. Polym. Ed..

[35] Bi Y.G., Lin Z.T., Deng S.T. (2019). Fabrication and characterization of hydroxyapatite/sodium alginate/chitosan composite microspheres for drug delivery and bone tissue engineering. Mater. Sci. Eng. C.

[36] Youssouf L., Bhaw-Luximon A., Diotel N., Catan A., Giraud P., Gimié F., Lallemand L. (2019). Enhanced effects of curcumin encapsulated in polycaprolactone-grafted oligocarrageenan nanomicelles, a novel nanoparticle drug delivery system. Carbohydr. Polym..

[37] Pozharitskaya O., Shikov A., Faustova N., Obluchinskaya E., Kosman V., Vuorela H., Makarov V. (2018). Pharmacokinetic and tissue distribution of fucoidan from *Fucus vesiculosus* after oral administration to rats. Mar. Drugs.

[38] Dhivya S., Padma V.V., Santhini E. (2015). Wound dressings—A review. BioMedicine.

[39] Pozharitskaya O.N., Shikov A.N., Obluchinskaya E.D., Vuorela H. (2019). The pharmacokinetics of fucoidan after topical application to rats. Mar. Drugs.

[40] O’Leary R., Rerek M., Wood E.J. (2004). Fucoidan modulates the effect of transforming growth factor (TGF)-β1 on fibroblast proliferation and wound repopulation in in vitro models of dermal wound repair. Biol. Pharm. Bull..

[41] Feki A., Bardaa S., Hajji S., Ktari N., Hamdi M., Chabchoub N., Amara I.B. (2019). Falkenbergia rufolanosa polysaccharide-poly (vinyl alcohol) composite films: A promising wound healing agent against dermal laser burns in rats. Int. J. Biol. Macromol..

[42] Slima S.B., Trabelsi I., Ktari N., Bardaa S., Elkaroui K., Bouaziz M., Salah R.B. (2019). Novel *Sorghum bicolor* (L.) seed polysaccharide structure, hemolytic and antioxidant activities, and laser burn wound healing effect. Int. J. Biol. Macromol..

[43] Janarthanan M., Senthil Kumar M. (2019). Extraction of alginate from brown seaweeds and evolution of bioactive alginate film coated textile fabrics for wound healing application. J. Ind. Text..

[44] Sellimi S., Maalej H., Rekik D.M., Benslima A., Ksouda G., Hamdi M., Hajji M. (2018). Antioxidant, antibacterial and in vivo wound healing properties of laminaran purified from Cystoseira barbata seaweed. Int. J. Biol. Macromol..

[45] Obluchinsksya E.D., Makarova M.N., Pozharitskaya O.N., Shikov A.N. (2015). Effects of ultrasound treatment on the chemical composition and anticoagulant properties of dry fucus extract. Pharm. Chem. J..

[46] Chen X., Song L., Wang H., Liu S., Yu H., Wang X., Li P. (2019). Partial characterization, the immune modulation and anticancer activities of sulfated polysaccharides from filamentous microalgae *Tribonema* sp.. Molecules.

[47] Alboofetileh M., Rezaei M., Tabarsa M. (2019). Enzyme-assisted extraction of Nizamuddinia zanardinii for the recovery of sulfated polysaccharides with anticancer and immune-enhancing activities. J. Appl. Phycol..

[48] Prabhu R., Mohammed M.A., Anjali R., Archunan G., Prabhu N.M., Pugazhendhi A., Suganthy N. (2019). Ecofriendly one pot fabrication of methyl gallate@ ZIF-L nanoscale hybrid as pH responsive drug delivery system for lung cancer therapy. Process Biochem..

[49] Ale M.T., Maruyama H., Tamauchi H., Mikkelsen J.D., Meyer A.S. (2011). Fucose-containing sulfated polysaccharides from brown seaweeds inhibit proliferation of melanoma cells and induce apoptosis by activation of caspase-3 in vitro. Mar. Drugs.

[50] Kyung J., Kim D., Park D., Yang Y.H., Choi E.K., Lee S.P., Kim Y.B. (2012). Synergistic anti-inflammatory effects of *Laminaria japonica* fucoidan and *Cistanche tubulosa* extract. Lab. Anim. Res..

[51] Senthilkumar K., Manivasagan P., Venkatesan J., Kim S.K. (2013). Brown seaweed fucoidan: Biological activity and apoptosis, growth signaling mechanism in cancer. Int. J. Biol. Macromol..

[52] Abudabbus A., Badmus J.A., Shalaweh S., Bauer R., Hiss D. (2017). Effects of fucoidan and chemotherapeutic agent combinations on malignant and non-malignant breast cell lines. Curr. Pharm. Biotechnol..

[53] Zhang C., Wang C., Tang S., Sun Y., Zhao D., Zhang S., Xiao X. (2013). TNFR1/TNF-α and mitochondria interrelated signaling pathway mediates quinocetone-induced apoptosis in HepG2 cells. Food Chem. Toxicol..

[54] Arumugam P., Arunkumar K., Sivakumar L., Murugan M., Murugan K. (2019). Anticancer effect of fucoidan on cell proliferation, cell cycle progression, genetic damage and apoptotic cell death in HepG2 cancer cells. Toxicol. Rep..

[55] Vaikundamoorthy R., Krishnamoorthy V., Vilwanathan R., Rajendran R. (2018). Structural characterization and anticancer activity (MCF7 and MDA-MB-231) of polysaccharides fractionated from brown seaweed Sargassum wightii. Int. J. Biol. Macromol..

[56] Schmidt C.E., Leach J.B. (2003). Neural tissue engineering: Strategies for repair and regeneration. Annu. Rev. Biomed. Eng..

[57] Yu X., Bellamkonda R.V. (2003). Tissue-engineered scaffolds are effective alternatives to autografts for bridging peripheral nerve gaps. Tissue Eng..

[58] Khan F., Ahmad S.R. (2013). Polysaccharides and their derivatives for versatile tissue engineering application. Macromol. Biosci..

[59] Lalzawmliana V., Anand A., Mukherjee P., Chaudhuri S., Kundu B., Nandi S.K., Thakur N.L. (2019). Marine organisms as a source of natural matrix for bone tissue engineering. Ceram. Int..

[60] Cho Y.S., Jung W.K., Kim J.A., Choi I.W., Kim S.K. (2009). Beneficial effects of fucoidan on osteoblastic MG-63 cell differentiation. Food Chem..

[61] Park S.B., Chun K.R., Kim J.K., Suk K., Jung Y.M., Lee W.H. (2010). The differential effect of high and low molecular weight fucoidans on the severity of collagen-induced arthritis in mice. Phytother. Res..

[62] Irhimeh M.R., Fitton J.H., Lowenthal R.M. (2007). Fucoidan ingestion increases the expression of CXCR4 on human CD3^4+^ cells. Exp. Hematol..

[63] Changotade S.I.T., Korb G., Bassil J., Barroukh B., Willig C., Colliec-Jouault S., Senni K. (2008). Potential effects of a low-molecular-weight fucoidan extracted from brown algae on bone biomaterial osteoconductive properties. J. Biomed. Mater. Res. Part A.

[64] Jin G., Kim G.H. (2011). Rapid-prototyped PCL/fucoidan composite scaffolds for bone tissue regeneration: Design, fabrication, and physical/biological properties. J. Mater. Chem..

[65] Lee J.S., Jin G.H., Yeo M.G., Jang C.H., Lee H., Kim G.H. (2012). Fabrication of electrospun biocomposites comprising polycaprolactone/fucoidan for tissue regeneration. Carbohydr. Polym..

[66] Barralet J.E., Wang L., Lawson M., Triffitt J.T., Cooper P.R., Shelton R.M. (2005). Comparison of bone marrow cell growth on 2D and 3D alginate hydrogels. J. Mater. Sci. Mater. Med..

[67] Turco G., Marsich E., Bellomo F., Semeraro S., Donati I., Brun F., Paoletti S. (2009). Alginate/hydroxyapatite biocomposite for bone ingrowth: A trabecular structure with high and isotropic connectivity. Biomacromolecules.

[68] Park D.J., Choi B.H., Zhu S.J., Huh J.Y., Kim B.Y., Lee S.H. (2005). Injectable bone using chitosan-alginate gel/mesenchymal stem cells/BMP-2 composites. J. Cranio Maxillofac. Surg..

[69] Li Z., Ramay H.R., Hauch K.D., Xiao D., Zhang M. (2005). Chitosan–alginate hybrid scaffolds for bone tissue engineering. Biomaterials.

[70] Lee G.S., Park J.H., Shin U.S., Kim H.W. (2011). Direct deposited porous scaffolds of calcium phosphate cement with alginate for drug delivery and bone tissue engineering. Acta Biomater..

[71] Yu C.C., Chang J.J., Lee Y.H., Lin Y.C., Wu M.H., Yang M.C., Chien C.T. (2013). Electrospun scaffolds composing of alginate, chitosan, collagen and hydroxyapatite for applying in bone tissue engineering. Mater. Lett..

[72] Nakaoka R., Hirano Y., Mooney D.J., Tsuchiya T., Matsuoka A. (2013). Study on the potential of RGD-and PHSRN-modified alginates as artificial extracellular matrices for engineering bone. J. Artif. Organs.

[73] Venkatesan J., Bhatnagar I., Kim S.K. (2014). Chitosan-alginate biocomposite containing fucoidan for bone tissue engineering. Mar. Drugs.

[74] Singh D., Zo S.M., Singh D., Han S.S. (2019). Interpenetrating alginate on gelatin–poly (2-hydroxyethyl methacrylate) as a functional polymeric matrix for cartilage tissue engineering. Int. J. Polym. Mater. Polym. Biomater..

[75] Ayoub A., Pereira J.M., Rioux L.E., Turgeon S.L., Beaulieu M., Moulin V.J. (2015). Role of seaweed laminaran from Saccharina longicruris on matrix deposition during dermal tissue-engineered production. Int. J. Biol. Macromol..

[76] Bhadja P., Tan C.Y., Ouyang J.M., Yu K. (2016). Repair effect of seaweed polysaccharides with different contents of sulfate group and molecular weights on damaged HK-2 cells. Polymers.

[77] Jung S.M., Kim S.H., Min S.K., Shin H.S. (2012). Controlled activity of mouse astrocytes on electrospun PCL nanofiber containing polysaccharides from brown seaweed. Vitro Cell. Dev. Biol. Anim..

[78] Popa E.G., Caridade S.G., Mano J.F., Reis R.L., Gomes M.E. (2015). Chondrogenic potential of injectable κ-carrageenan hydrogel with encapsulated adipose stem cells for cartilage tissue-engineering applications. J. Tissue Eng. Regen. Med..

[79] Farage M.A., Miller K.W., Elsner P., Maibach H.I. (2008). Intrinsic and extrinsic factors in skin ageing: A review. Int. J. Cosmet. Sci..

[80] Matsumura Y., Ananthaswamy H.N. (2004). Toxic effects of ultraviolet radiation on the skin. Toxicol. Appl. Pharmacol..

[81] Moon H.J., Lee S.H., Ku M.J., Yu B.C., Jeon M.J., Jeong S.H., Lee Y.H. (2009). Fucoidan inhibits UVB-induced MMP-1 promoter expression and down regulation of type I procollagen synthesis in human skin fibroblasts. Eur. J. Dermatol..

[82] Polefka T.G., Meyer T.A., Agin P.P., Bianchini R.J. (2012). Effects of solar radiation on the skin. J. Cosmet. Dermatol..

[83] Fuentealba D., Galvez M., Alarcon E., Lissi E., Silva E. (2007). Photosensitizing activity of advanced glycation endproducts on tryptophan, glucose 6-phosphate dehydrogenase, human serum albumin and ascorbic acid evaluated at low oxygen pressure. Photochem. Photobiol..

[84] Priyan Shanura Fernando I., Kim K.N., Kim D., Jeon Y.J. (2019). Algal polysaccharides: Potential bioactive substances for cosmeceutical applications. Crit. Rev. Biotechnol..

[85] Shah H., Rawal Mahajan S. (2013). Photoaging: New insights into its stimulators, complications, biochemical changes and therapeutic interventions. Biomed. Aging Pathol..

[86] Kim J.A., Ahn B.N., Kong C.S., Kim S.K. (2013). The chromene sargachromanol E inhibits ultraviolet A-induced ageing of skin in human dermal fibroblasts. Br. J. Dermatol..

[87] Shao P., Chen X., Sun P. (2015). Improvement of antioxidant and moisture-preserving activities of *Sargassum horneri* polysaccharide enzymatic hydrolyzates. Int. J. Biol. Macromol..

[88] Li Z.Y., Yu C.H., Lin Y.T., Su H.L., Kan K.W., Liu F.C., Lin Y.H. (2019). The potential application of spring *Sargassum glaucescens* extracts in the moisture-retention of keratinocytes and dermal fibroblast regeneration after UVA-irradiation. Cosmetics.

[89] Ye Y., Ji D., You L., Zhou L., Zhao Z., Brennan C. (2018). Structural properties and protective effect of Sargassum fusiforme polysaccharides against ultraviolet B radiation in hairless Kun Ming mice. J. Funct. Foods.

[90] Moon H.J., Park K.S., Ku M.J., Lee M.S., Jeong S.H., Imbs T.I., Lee Y.H. (2009). Effect of Costaria costata fucoidan on expression of matrix metalloproteinase-1 promoter, mRNA, and protein. J. Nat. Prod..

[91] Fitton J., Dell’Acqua G., Gardiner V.A., Karpiniec S., Stringer D., Davis E. (2015). Topical benefits of two fucoidan-rich extracts from marine macroalgae. Cosmetics.

