# Supplementary material for: Marine Seaweed Polysaccharides-Based Engineered Cues for the Modern Biomedical Sector
Source: Mar Drugs. 2019 Dec 19;18(1):7. doi: 10.3390/md18010007 (PMC7024278; doi:10.3390/md18010007)
Supplement: Supplementary file 1 [file marinedrugs-18-00007-s001.pdf]

Supplementary Data:

Table S1. Literature search results obtained from the Scopus database. The distribution of articles in each search category is based on total number of articles and reads from top to bottom column wise.

Search Terms	Document Types	# of Articles from All Years		# of Articles from Top Journals		# of Articles Based on Territory	
Marine seaweed polysaccharides	Article, Review, Book Chapter, Conference Paper, Book	2019	43	*Marine Drugs*	41	India	62
		2018	46	*Journal of Applied Phycology*	15	South Korea	59
		2017	47	*Carbohydrate Polymers*	12	China	48
		2016	55	*International Journal of Biological Macromolecules*	12	Brazil	40
		2015	45	*Advances in Food and Nutrition Research*	11	France	34
		All past years	233	All other Journals	378	Other countries	226
Drug delivery using polysaccharides	Article, Review, Book Chapter, Conference Paper	2020	9	*Carbohydrate Polymers*	235	United States	874
		2019	441	*International Journal of Biological Macromolecules*	202	India	873
		2018	470	*Journal of Controlled Release*	171	China	849
		2017	455	*International Journal of Pharmaceutics*	157	Italy	311
		2016	409	*Biomaterials*	129	United Kingdom	289
		All past years	3433	All other Journals	4323	Other countries	2021
Wound healing using polysaccharides	Article, Review, Conference Paper, Book Chapter, Short Survey	2020	2	*International Journal of Biological Macromolecules*	60	United States	214
		2019	140	*Carbohydrate Polymers*	57	China	203
		2018	130	*Biomaterials*	30	India	137
		2017	117	*Biomacromolecules*	20	United Kingdom	80
		2016	89	*Journal of Ethnopharmacology*	16	Italy	66
		All past years	737	All other Journals	1032	Other countries	515
Anticancer activities of polysaccharides	Article, Review, Book Chapter, Conference Paper, Short Survey	2020	4	*Carbohydrate Polymers*	71	China	333
		2019	113	*International Journal of Biological Macromolecules*	71	India	127
		2018	126	*Marine Drugs*	22	South Korea	123
		2017	109	*International Journal of Medicinal Mushrooms*	15	United States	99
		2016	94	*Molecules*	13	Japan	43

		All past years	551	All other Journals	805	Other countries	272
Polysaccharide-based engineered carriers	Article, Review, Book Chapter	2019	3	*ACS Applied Materials and Interfaces*	1	France	5
		2018	3	*Accounts of Chemical Research*	1	United States	3
		2017	3	*American Journal of Cancer Research*	1	Australia	2
		2016	3	*Carbohydrate Polymers*	1	China	2
		2015	2	*Colloids and Surfaces B Biointerfaces*	1	Italy	2
		All past years	5	All other Journals	14	Other countries	5
